# Flexor digitorum brevis utilizes elastic strain energy to contribute to both work generation and energy absorption at the foot

**DOI:** 10.1242/jeb.243792

**Published:** 2022-04-22

**Authors:** Ross E. Smith, Glen A. Lichtwark, Luke A. Kelly

**Affiliations:** School of Human Movement and Nutrition Sciences, The University of Queensland, Brisbane, QLD 4072, Australia

**Keywords:** Intrinsic foot muscles, Foot biomechanics, Muscle dynamics, Foot energetics

## Abstract

The central nervous system utilizes tendon compliance of the intrinsic foot muscles to aid the foot's arch spring, storing and returning energy in its tendinous tissues. Recently, the intrinsic foot muscles have been shown to adapt their energetic contributions during a variety of locomotor tasks to fulfil centre of mass work demands. However, the mechanism by which the small intrinsic foot muscles are able to make versatile energetic contributions remains unknown. Therefore, we examined the muscle–tendon dynamics of the flexor digitorum brevis during stepping, jumping and landing tasks to see whether the central nervous system regulates muscle activation magnitude and timing to enable energy storage and return to enhance energetic contributions. In step-ups and jumps, energy was stored in the tendinous tissue during arch compression; during arch recoil, the fascicles shortened at a slower rate than the tendinous tissues while the foot generated energy. In step-downs and landings, the tendinous tissues elongated more and at greater rates than the fascicles during arch compression while the foot absorbed energy. These results indicate that the central nervous system utilizes arch compression to store elastic energy in the tendinous tissues of the intrinsic foot muscles to add or remove mechanical energy when the body accelerates or decelerates. This study provides evidence for an adaptive mechanism to enable the foot's energetic versatility and further indicates the value of tendon compliance in distal lower limb muscle–tendon units in locomotion.

## Abstract (precis)

**Summary:** Demonstration of an adaptive mechanism that enables the intrinsic foot muscles to make versatile contributions to whole-body accelerations and decelerations.

## INTRODUCTION

The human foot is known to possess spring-like compliance in its long arch, which allows energy savings and aids in propulsion during locomotion (Ker et al., 1987; Stearne et al., 2016). The plantar aponeurosis, a thick, ligamentous connective tissue sheath along the foot's plantar surface, makes passive contributions to the foot's arch-spring by absorbing and returning energy over the course of gait stance (McDonald et al., 2016; Welte et al., 2021). The intrinsic foot muscles also span the length of the long arch and have been suggested to contribute to the foot's arch-spring mechanism (Kelly et al., 2019). Additionally, the intrinsic foot muscles actively modulate the foot's mechanics to meet energetic requirements during tasks requiring rapid accelerations and decelerations (Smith et al., 2021). Therefore, the intrinsic foot muscles (IFM) are thought to enable the foot's functional versatility during a range of locomotive modalities.

To accelerate or decelerate the body during locomotion, muscles act to generate or dissipate mechanical energy. The ankle plantar flexors are the largest average power contributors to walking and running (Farris and Sawicki, 2012), and a substantial amount of the power contributed comes from the elastic tendinous tissue (Lichtwark and Wilson, 2006). This is possible because of the interaction between the short-pennate muscle fibres of the gastrocnemii and soleus and the Achilles tendon. For example, during the first half of stance phase in locomotion, dorsiflexion of the ankle joint results in elongation of the muscle–tendon unit (MTU), while the fascicles of the plantar flexors remain quasi-isometric, facilitating stretch of the elastic tendon (Fukunaga et al., 2001; Lichtwark and Wilson, 2006; Lichtwark et al., 2007). Throughout this period, neural drive to the ankle plantar flexors is controlled so that force produced by the muscles is sufficient to maintain a quasi-isometric contraction, allowing strain energy to be stored in the stretching tendon. During propulsion, the energy stored in the tendon is returned to the body via tendon recoil, delivering mechanical power to propel the body forward (Fukunaga et al., 2001; Lichtwark et al., 2007).

Decoupling the MTU and tendon length changes from the contractile length change also enables stored energy to be returned through MTU recoil while the fascicles actively shorten, allowing supramaximal power outputs from the ankle plantar flexors when generating energy during accelerations (Kurokawa et al., 2003; Farris et al., 2016; Wade et al., 2018). However, energy storage prior to enhanced energy generation is performed by muscular work through fascicle shortening, such that the energy stored in the Achilles tendon during ankle dorsiflexion is subsequently returned by the tendon at a greater velocity than is achieved by its fascicles during propulsion, acting to ‘amplify’ plantar flexor power outputs (Roberts and Azizi, 2011). When decelerating the body, the ankle plantar flexor's MTU lengthen significantly more than the fascicles, acting as a velocity buffer from high-power inputs into muscles (Werkhausen et al., 2017). For this to occur, the initial high-velocity elongation of the MTU is largely attributed to tendon elongation, rather than actively lengthening muscle fibres. Subsequently, as force and velocity decline, the tendon recoils and the muscle fibres actively lengthen to dissipate the energy that is stored in the stretched tendon. This mechanism delays and extends the period over which the muscle dissipates energy, potentially protecting the muscle tissue from excessively high strains and forces (Werkhausen et al., 2017). Therefore, the architectural arrangement of muscles with short fibres and a long compliant tendon at the ankle joint enables the functional versatility required beyond steady-state locomotion.

In a series of recent experiments, we have shown that during stepping, jumping and landing, the central nervous system adapts the energetic behaviour of the foot to perform net work based on centre of mass (COM) work demands (Riddick et al., 2019; Smith et al., 2021). In these experiments, 8–17% of the mechanical work performed at the COM is contributed by structures within the foot. We have also shown that if we remove active force production within the foot by anaesthetizing the IFM, the energetic contributions of the foot to COM work decrease by ∼3% during running, jumping and landing (Farris et al., 2019; Smith et al., 2021). These findings indicate that the IFM are directly linked to the foot's functional versatility and capacity to dissipate and generate mechanical energy. However, the mechanisms for dissipating or generating significant amounts of energy when the IFM [e.g. flexor digitorum brevis (FDB) or abductor hallucis (AH)] possess relatively short muscle fibres is currently unknown. A recent study has demonstrated that the FDB facilitates elastic storage and return in its tendonous tissues during cyclical tasks designed to emulate the loading demands of steady-state locomotion (Kelly et al., 2019). Therefore, it is possible that the IFM may utilize their elastic elements to enhance energy generation and dissipation capacity within the foot; however, this has yet to be explored.

Here, we examined the dynamic behaviour of the FDB MTU and muscle fascicles during tasks where mechanical energy was generated and dissipated at the foot. We hypothesized that FDB muscle–tendon behaviours and activity will display specific behaviours based on the energetic function of the foot and the intensity of the task (increased foot power generation or absorption) such that: (1) during foot energy generation, the FBD fascicles would have reduced lengthening magnitude and velocity relative to the MTU during initial loading of the foot (suggesting energy storage in FDB tendinous tissue) and would actively shorten during propulsion, but with lower velocity than the MTU (suggesting energy release from FDB tendinous tissue), and that this effect would be greater with increased task intensity; and (2) during foot energy dissipation, FBD muscle fascicles would lengthen with lower magnitude and velocity than the MTU (suggesting that energy is stored in FDB tendinous tissue) and that this effect would be greater with increased task intensity.

## MATERIALS AND METHODS

### Participants

Fourteen (12 male, 2 female, 26.2±14.8 years, 1.72±0.23 m, 73.9±15.9 kg, means±s.d.) participants were recruited in accordance with an *a priori* analysis (G-power, version 3.1.9.2) powered at β=0.90 for a large effect of *d*=1 and *a*=0.05. Data from one subject were excluded as their ultrasound fascicle data were of too low a quality to measure accurately (*n*=13 for all analyses). Participants were 18–45 years old, were recreationally active (able to walk and run for 30 min consecutively) and had no lower limb injuries within 12 months of data acquisition. The University of Queensland's Human Research Ethics Committee approved this research protocol and ensured it operated in accordance with the Declaration of Helsinki. Written informed consent was obtained from all participants.

### Experimental task

**Fig. 1.**
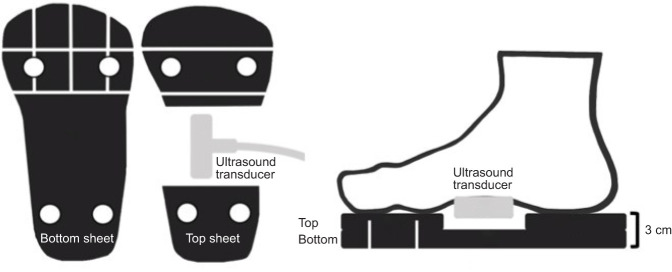
**Foam shoe design to house the ultrasound probe during all experimental tasks.** Foam shoe from the superior (left) and medial (right) aspect. Part of the midfoot region of the top sheet of foam was cut away to house the ultrasound probe. Three longitudinal and two transverse incisions were made in the foam to maximize the flexibility of the shoe and enable natural motion of the forefoot and toes during the experimental tasks. These incisions are depicted as white lines in each image.

Participants performed two energy generation tasks (step-up or jump) and two energy dissipation tasks (step-down or landing). All tasks were performed unilaterally to ensure no compensatory efforts from the non-studied limb. The energy generation tasks involved stepping and jumping from a force plate on the ground onto a 20.5 cm box located in front of the participants. A piece of tape was placed 1 cm from the edge of the force plate closest to the box. The most distal portion of each participant's foot was aligned with this tape before each trial, ensuring similar step or jump distances in the fore–aft direction. For the energy dissipation tasks, participants were asked to step-down or drop from the same 20.5 cm box onto a force plate in front of the box. The box was placed 5 cm from a piece of tape on the force plate, which was used as a stepping or landing target for the heel. The order of energy generation or dissipation tasks was randomized. Before collection trials both with and without the custom foam shoe (discussed below), participants were allowed to perform familiarization trials, which were simply practice trials for all tasks. No data were collected and these trials were performed to ensure correct task execution and participant comfort. Following completion of all familiarization tasks, an ultrasound transducer was secured with a cohesive bandage to the plantar surface of the dominant foot, over the muscle belly of the FDB as previously described (Kelly et al., 2019). Foot dominance was determined by asking participants which foot they would kick a ball with. After securing the transducer and ensuring ultrasound image quality, a participant-specific custom foam shoe was secured to the foot to protect the transducer from damage and to maintain reasonably constant contact pressure of the probe against the foot (Fig. 1). After fitting of the shoe, participants then repeated the protocol detailed above. All participants were encouraged to participate in additional familiarization trials for all tasks after the fitting of the shoe to ensure they were comfortable with task performance.

To perform the step-up trial, participants lifted their non-dominant foot onto the box while pushing off from the force plate with their dominant foot in order to propel themselves onto the box. Participants were cued to make contact with the box on their non-dominant foot only after the dominant foot's heel was off the ground to encourage push-off power generation by the stance foot. During the jumping task, participants jumped from their dominant foot onto the box, landing on both feet to ensure safety. Participants were discouraged from swinging the non-dominant leg to generate energy for upward propulsion of the COM. However, because of balance constraints from the unilateral task, use of the arms and arm swing were permitted.

For the step-down trials, participants stepped down from the box onto their dominant foot, and were cued to limit use of the non-dominant leg to lower the COM. For drop landings, participants dropped from the box onto their dominant foot. A successful landing trial was one where participants leaned anteriorly until they began to fall, then immediately lifted their non-dominant leg off of the box and landed on their dominant foot. This method has been described previously by the cue ‘walking the plank’ (Smith et al., 2021) and was used to discourage lowering of the COM by the non-dominant limb before initiation of the drop.

### Custom foam shoe

**Fig. 2.**
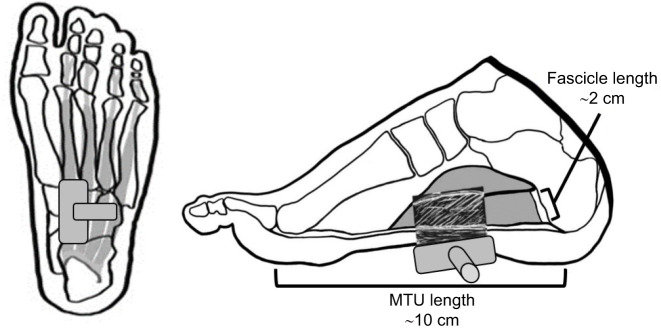
**Orientation of the ultrasound transducer.** The ultrasound probe, flexor digitorum brevis (shaded grey) and nearby foot anatomy from the inferior (left) and medial (right) aspect. In the medial aspect, a typical example of an ultrasound image for the flexor digitorum brevis is depicted to aid in visualizing the muscle's architecture, and muscle and total muscle–tendon unit (MTU) lengths from Kura et al. (1997) and Ledoux et al. (2001) are provided.

All foam shoes followed the same design but were customized to fit each participant. The shoes were made from two sheets of Shore A45 ethylene vinyl acetate foam (15 mm thickness, MetroFoam, Silverwater, NSW, Australia), glued together with contact adhesive. This foam hardness was chosen to minimize energy absorption/dissipation within the foam, whilst retaining comfort. The bottom sheet (Fig. 1) was cut to a generic shoe shape and sized to each participant. The top sheet was cut as a copy of the bottom sheet with a gap between the heel and forefoot sections to house the ultrasound transducer (Fig. 1). Incisions were made in the foam to reduce the bending stiffness of the shoe and facilitate natural motion of the arch and metatarsalphalangeal (MTP) joints. Fig. 2 depicts the orientation of the probe against the foot's plantar surface in order to obtain a satisfactory ultrasound image.

### Data processing and analysis

#### Kinematics and kinetics

3D motion-capture data were collected for all experimental tasks at 250 Hz (Oqus, Qualisys, Gothenburg, Sweden). To quantify motion of the pelvis and lower limb, reflective markers (9 mm, B&L Engineering, Santa Ana, CA, USA) were placed on the left and right posterior superior iliac crests and anterior superior iliac crests to define the pelvis segment; on the medial and lateral epicondyles to define the thigh segment; and on the medial and lateral malleoli to define the shank segment. Additional 4-marker rigid clusters were secured to the thigh and shank segments to track motion of their respective segments. To establish individual foot segments and track ankle and foot motion during the experimental trials, reflective markers were also placed on the peroneal tubercle, sustentaculum tali and calcaneal tuberosity to define the calcaneus; on the medial aspect of the first metatarsal base and head, superior aspect of the second metatarsal base and head, and lateral portion of the fifth metatarsal base and head to define the metatarsal segment; and on the medial aspect of the proximal first phalanx, and the superior aspects of the second and fourth intermediate phalanges to establish a toe segment in accordance with previous literature (Leardini et al., 2007; Kelly et al., 2015).

A static calibration trial was captured to establish segment dimensions, and a six degrees of freedom model was constructed for the calculation of joint kinematics and kinetics. Using Visual 3D (C-Motion, Inc., Germantown, MD, USA), data were low-pass filtered with a second-order bi-directional Butterworth filter at 25 Hz. Joint rotation was expressed about their proximal reference segment. Ankle joint rotation was expressed as rotation of the calcaneus about the shank, while deformation of the longitudinal arch (LA) was quantified as sagittal plane rotation of the metatarsal segment relative to the calcaneus. Rotational polarity of all joints and their Cardan rotational sequences were assigned using a right-hand rule with *x* as the longitudinal axis, *y* as the anteroposterior axis, and *z* as the mediolateral axis for tri-planar calculations.

Ground reaction force data were collected at 2500 Hz (AMTI, Watertown, MA, USA) and filtered in Visual3D using the same frequency cut-off to avoid the possibility of sagittal plane moment artefacts (Derrick et al., 2020). Using filtered force data and joint kinematic data, net internal joint moments were calculated using a Newtonian–Euler inverse dynamics solution within Visual3D software. Relative segment mass, joint centres, COM location and moments of inertia were based on previous literature (Dempster, 1955; Hanavan, 1964). Foot power was calculated using a unified deformable (UD) segment analysis that is described in previous literature (Takahashi et al., 2012, 2016). The UD segment analysis models the foot as a hybrid segment which includes a rigid, proximal component and a distal, deformable component (Takahashi et al., 2012). It provides an estimate of the power of all structures distal to the calcaneus (i.e. fat pads, plantar ligaments, etc.) and has previously been used to describe the foot's gross energetic function (Riddick et al., 2019; Smith et al., 2021). Foot work was calculated as trapezoidal integration of the UD foot power metric.

For step-up data analyses, time-series data waveforms begin at the first 10% deviation from bodyweight of the vertical ground reaction force component, through to take-off. For the jump analyses, time-series data begin at countermovement initiation and continue through to take-off, where countermovement initiation was defined as the point at which pelvis-segment velocity decreased below a −0.075 m s^−1^ threshold (Smith et al., 2021). This was chosen in order to account for oscillations in pelvis velocity during unilateral stance. Data used in statistical comparisons were taken from phases in time-series data from the point at which FDB MTU lengths began to shorten by more than 0.1 mm for two consecutive frames until take-off (propulsion start). For both step-downs and landings, time-series data were taken from foot contact until pelvis velocity was no longer negative. Data used in statistical comparisons were taken from highlighted phases in time-series data from the point at which FDB MTU lengths were lengthening until length change ceased to exceed 0.1 mm for five consecutive frames (0.02 s) (deceleration end). For all tasks, time-series data are presented as total movement time normalized to 101 points.

#### Muscle–tendon dynamics and activation

B-mode ultrasound imaging for the FDB was collected using a custom-built T-shaped 128-element, 4 cm ultrasound transducer (LZ 7.5/60/128Z-2, Telemed, Vilnius, Lithuania). The transducer was placed over the FDB muscle belly, in line with the second metatarsal and secured with self-adhesive tape (Kelly et al., 2019). Capture frame rate ranged from 134 to 171 Hz and varied based on changes in settings within Telemed software that were made to optimize the image for data analysis. Muscle fascicle data were processed using a semi-automated optic affine flow algorithm to measure the movement of muscle fascicles and apply the observed displacement of the muscle to defined distal and proximal endpoints from frame to frame (Farris and Lichtwark, 2016; Cronin et al., 2011). According to a model used previously (Kelly et al., 2015, 2019), FDB MTU length data were calculated from kinematic data as the distance between the muscle model's origin at the calcaneus, to its insertion at the phalanges about a tethering point at the MTP joint. Mean fascicle and MTU velocity were calculated as the first derivative of FDB fascicle or MTU length with respect to time. For all tasks, positive MTU and fascicle velocities indicate lengthening velocities and negative values indicate shortening velocities.

Muscle activation data (EMG) for the FDB were sampled at 4000 Hz, amplified ×1000, and recorded at a 30–1000 bandwidth (MA300, Motion Lab Systems, Baton Rouge, LA, USA). Fine-wire electrodes (0.051 mm stainless steel, Teflon coated, Chalgren, Columbia, SC, USA) were inserted into the FDB muscle under ultrasound guidance (10 MHz linear array, SonixTouch, Ultrasonix, BC, Canada) in accordance with previously described methods (Kelly et al., 2012, 2015, 2018). The EMG data were processed using a custom MATLAB (MathWorks, Natick, MD, USA) script that: removed any DC offset; applied a second-order Butterworth high-pass filter at 50 Hz; rectified the signal; low-pass filtered the signal at 10 Hz for data smoothing; and normalized to the maximum rectified signal amplitude recorded across all tasks. Thus, EMG signals are presented as a percentage of peak activation, where peak activation was the largest activation value across all tasks.

#### Statistics

A two-way (muscle level×task intensity), within-subjects, repeated measures ANOVA (Jamovi, version 1.6, www.jamovi.org) was performed for both energy generation and dissipation tasks to assess whether there were differences in MTU and fascicle velocity (muscle level) and whether this was influenced by task velocity constraints (task intensity). *Post hoc* multiple comparisons *t*-tests were performed to detect the nature of any interaction effects, and Bonferroni corrections were made to reduce the likelihood of type 1 error with multiple comparisons. Additionally, paired-samples *t*-tests were performed to assess the effect of increased task velocity on peak power and work performed at the foot, as well as mean and peak FDB EMG. Statistical significance for all statistical tests was set at *P*≤0.05. All results are reported as means±s.d.

## RESULTS

### Energy generation

#### MTU and fascicle behaviour

**Fig. 3.**
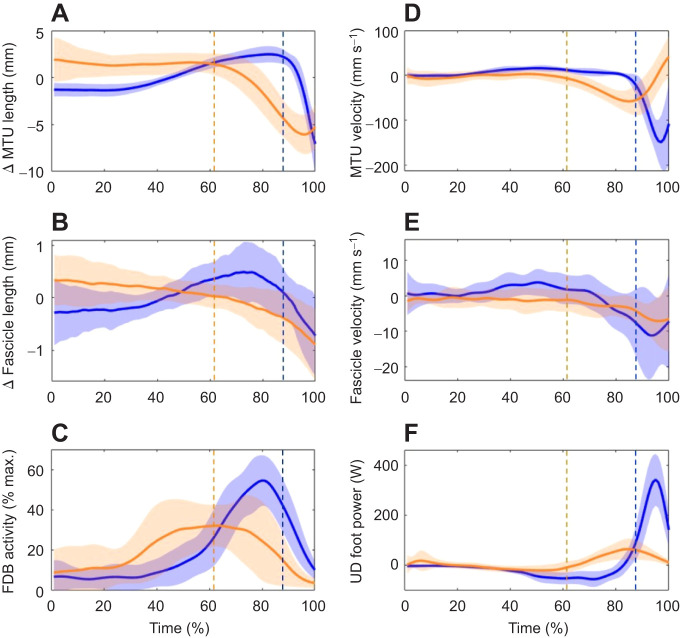
**Group mean ensemble data obtained during the energy generation tasks.** (A) Change in MTU length, (B) change in fascicle length, (C) flexor digitorum brevis (FDB) rectified muscle activity, (D) MTU velocity, (E) fascicle velocity and (F) unified deformable (UD) foot power over time. Data from the step-ups (orange) and jumps (blue) were time normalized over the defined movement phases. Dashed vertical lines at 61% of normalized movement time (step-ups) and 89% of normalized movement time (jumps) represent the start of the FDB MTU shortening phase.

Fig. 3A,B shows FDB MTU and fascicle length changes during step-ups and jumps. The MTU length changes for both tasks (step-ups ∼8 mm; jumps ∼10 mm) were much larger than fascicle length changes (step-ups ∼0.9 mm, jumps ∼0.7 mm). We also observed differences in the timing of length changes occurring at the MTU and the fascicles between step-ups and jumps. During step-ups, the fascicles began shortening as early as 10% of total movement time and continued shortening until take-off, whereas the MTU began shortening at ∼60% of total movement time. During jumps, fascicle shortening was preceded by a phase of slight lengthening from ∼30% to 75% of total movement time, after which the fascicles shortened until take-off. MTU length changes were larger by an order of magnitude and showed a clear period of lengthening from 30% to 90% of total movement time, followed by rapid shortening prior to take-off. During both tasks, FDB fascicle shortening preceded FDB MTU shortening, further highlighting the dissociation in the dynamic behaviour of the contractile and tendinous tissues. FDB MTU and fascicle velocities are shown in Fig. 3D,E. We observed main effects for muscle level (MTU versus fascicle, *P*<0.001) and task intensity (step-ups versus jumps, *P*<0.001), as well as an interaction effect (*P*<0.001). *Post hoc t*-tests revealed that while mean fascicle shortening velocities were faster during jumps (−9.83±7.6 mm s^−1^) than during step-ups (−3.81±3.1 mm s^−1^, *P*=0.039), the interaction effect was largely driven by the substantially higher MTU shortening velocities during jumps (−106.92±36.7 mm s^−1^) compared with step-ups (−38.6±11.1 mm s^−1^, *P*<0.001).

#### Muscle activation

The FDB EMG signals also displayed differences in the timing of activation between tasks (Fig. 3C). During step-ups, muscle activation increased relatively quickly (19–41% of maximum), peaked at ∼50% of total movement time and began to decay at ∼75% of total movement time. During jumps, activation increased from ∼35% to peak at ∼80% of total movement time, after which it decayed. Neither mean (step-ups: 23.2±12.68%; jumps: 25.3±6.47%, *P*=0.419) nor peak FDB EMG (step-ups: 39.6±20%; jumps: 44.25±8.79%, *P*=0.375) differed between step-ups and jumps.

#### Foot energetics

Peak power generation (step-ups 73.2±38 W, jumps 380±133.2 W) and positive work (step-ups 9.1±4.95 J, jumps 20.8±8.47 J) at the foot were greater during jumps than during step-ups (both *P*<0.001). The primary burst of positive power at the foot occurred concurrently with the periods of peak MTU and fascicle shortening velocity (Fig. 3F), indicating recoil of the MTU contributes positive power at the foot.

### Energy dissipation

#### MTU and fascicle behaviour

**Fig. 4.**
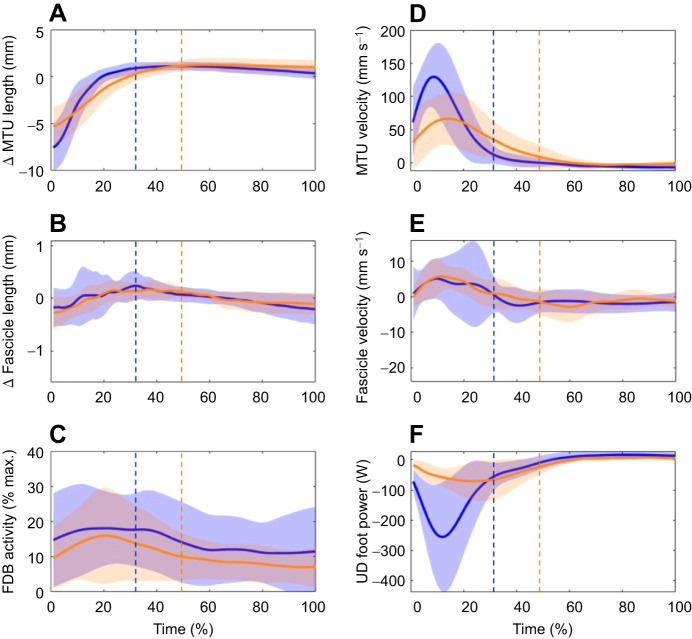
**Group mean ensemble data obtained during the energy dissipation tasks.** (A) Change in MTU length, (B) change in fascicle length, (C) FDB rectified muscle activity, (D) MTU velocity, (E) fascicle velocity and (F) UD foot power over time. Data from the step-downs (orange) and landings (blue) were time normalized over the defined movement phases. Dotted vertical lines at 48% of normalized time (step-downs) and 33% of normalized time (landings) represent the start of the FDB MTU lengthening phase.

FDB MTU and fascicle length changes for step-downs and landings are shown in Fig. 4A,B. The MTU underwent an initial lengthening after foot contact, followed by a plateau (dissipation end) during both step-down (33% of total movement time) and landing (48% of total movement time) tasks. The FDB fascicles lengthened slightly, but generally functioned quasi-isometrically during both tasks. For both tasks, the FDB MTU length change was substantially larger than the fascicle length change (step-downs: MTU ∼6 mm, fascicles ∼0.4 mm; landings: MTU ∼9 mm, fascicles ∼0.4 mm). The FDB MTU and fascicle velocities are shown in Fig. 4D,E. We observed main effects for muscle level (MTU versus fascicle, *P*<0.001) and task intensity (step-down versus landings, *P*<0.001), as well as an interaction effect (*P*<0.001). *Post hoc* analyses revealed the interaction effect was driven by the increase in MTU lengthening velocity during the landings compared with the step-downs (step-downs: 54.7±25.8 mm s^−1^, landings: 98.3±43.2 mm s^−1^; *P*<0.001) and not by any changes in fascicle velocity between conditions (step-downs: 3.85±3.31 mm s^−1^, landings: 6.05±5.49 mm s^−1^; *P*=0.234).

#### Muscle activation

The FDB EMG during the tasks is shown in Fig. 4C. The muscle was active prior to foot contact and remained active at a similar percentage of peak activation throughout the energy absorption phase for step-downs (9–15% of maximum) and landings (12–18% of maximum). There were no statistically significant differences in mean (step-downs: 14.6±10.59%, landings: 17±11.64%; *P*=0.413) or peak (step-downs: 19.5±14.31%, landings: 21.4±13.74%, *P*=0.644) EMG between step-downs and landings.

#### Foot energetics

Peak power absorption (step-downs −12.3±12.7 W, landings −55.45±40.6 W; *P*=0.002) and negative work (step-downs −6.9±3.84 J, landings −14.9±10.47 J; *P*=0.004) at the foot were reduced during step-downs compared with landings. During step-downs and landings, the peak in negative foot power following foot–ground impact occurred concurrently with the peak in MTU lengthening velocity (Fig. 4F).

## DISCUSSION

Compression and recoil of the long arch in the foot allows energy storage and return via elastic stretch and recoil of the plantar aponeurosis, ligaments and muscles that span this structure (Ker et al., 1987; Stearne et al., 2016; Kelly et al., 2019). In this study, we have shown that human long arch mobility can also be exploited during whole-body accelerations and decelerations to add or remove mechanical energy from the body. This is facilitated via a control scheme that decouples muscle fascicle and MTU lengthening behaviour, utilizing energy storage in the tendinous tissues of the intrinsic foot muscles. When mechanical power output and input to the foot increased, the disparity between MTU and fascicle length changes and velocities increased, indicating greater utilization of the tendinous tissues to amplify or buffer power output and input, with increasing demand. In addition to the known contribution of the passive elastic tissues within the arch of the foot (Ker et al., 1987; Stearne et al., 2016), this reveals an adaptive mechanism providing versatility for the foot to perform a myriad of locomotor tasks.

During the countermovement phase of the jump task, the MTU lengthened as the longitudinal arch compressed (lengthened and lowered), while the fascicles actively lengthened very slightly (∼1 mm), but generally remained quasi-isometric. This suggests that the FDB muscle is storing energy in the tendinous tissues during the countermovement, allowing force to be produced (and energy to be stored) over a longer period of time. Once MTU shortening had begun, both the MTU and fascicles shortened until toe-off in both tasks. Given that MTU shortening velocity was substantially faster than fascicle shortening velocity in the jumping condition, these data indicate the FDB muscle can utilize elastic energy during MTU recoil to generate power during propulsion. While the step-up task did not require a countermovement, the same pattern of decoupling was evident in the early phase of the movement, with fascicles shortening for an extended period of time, prior to any length change occurring in the MTU. Previous research in the human gastrocnemius during jumping has demonstrated that similar MTU behaviour allows supramaximal power output, such that the stored elastic energy in the tendon adds to net energy generated by the fascicles and is returned at a faster rate than the fascicles could produce (Kurokawa et al., 2003; Farris et al., 2016). In our data, the period of MTU recoil coincided with a relatively large increase in foot power in jumping compared with step-ups, indicating the FDB fascicles utilize tendon elasticity to increase net power output from the MTU.

During the energy dissipation tasks, the MTU length changes and velocities were an order of magnitude greater than those occurring in the muscle fascicles. When considered in context of the large magnitudes of energy dissipated at the foot during these tasks, this decoupling highlights the use of the tendinous tissues for energy absorption during the impact period. These findings for the FDB resemble the MTU dynamics of the lateral gastrocnemius of humans (Werkhausen et al., 2017) and wild turkeys (Konow et al., 2012; Konow and Roberts, 2015) during landing tasks. However, the decline in magnitude of the ground reaction force (force decay) sees this stored energy in the gastrocnemius returned to the muscles via tendon recoil while the fascicles actively lengthen, dissipating the energy stored in the tendon (Konow et al., 2012; Konow and Roberts, 2015; Werkhausen et al., 2017). Here, the FDB fascicles shortened during force decay with very little tendon recoil, perhaps indicating the energy remained stored in the tendon during subsequent weight bearing (and perhaps becomes dissipated by the muscle when the foot is unloaded) or that the energy was dissipated elsewhere. However, an analysis over a longer period of time would be necessary to resolve this. The large differences in MTU velocity relative to fascicle velocity in our data suggest that the FDB tendon acts as a buffer for the contractile tissues, sparing the muscle from experiencing high and potentially damaging strains and strain rates during rapid decelerations of the body (Konow et al., 2012; Konow and Roberts, 2015).

There is increasing evidence that the stiffness of the human foot is affected by more than the interaction between the longitudinal arch and the plantar fascia. Recently, the unique transverse curvature of the human foot has been revealed to play a part in the foot's stiffness (Venkadesan et al., 2020). The shape of the human foot is likely to be an important feature that enables considerable foot mobility, with the human midfoot actually displaying substantially more motion than that observed in non-human primates during locomotion (Holowka et al., 2017). We believe it is this unique osseous morphology combined with the muscle architecture and passive elastic tissues that enables such versatility in human foot function, as highlighted in a recent publication from our laboratory (Smith et al., 2021). There is evidence for differences in foot muscle architecture across hominoids (Oishi, et al., 2009; Oishi et al., 2018), with fascicle lengths seemingly longer in gorillas, orangutans, chimpanzees and bonobos than in humans. Longer fascicles (relative to muscle–tendon unit length) are better suited to generating joint excursion and power (Biewener and Roberts, 2000; Roberts and Azizi, 2011) and less suited to storage and return of elastic energy. As such, it is likely that, relative to those of other apes and hominids, the human foot is well adapted to utilize elastic energy from the intrinsic foot muscles, providing the versatility required for habitual upright locomotion. (Kelly et al., 2018; Riddick et al., 2019; Smith et al., 2021).

Our findings must be considered amidst the experimental limitations. All fascicle images were taken from the compartment of the FDB in line with the 2nd metatarsal. Therefore, our results may be over-extrapolating the behaviour of the FDB muscle's other three compartments. Additionally, despite the similarities in muscle and tendon length ratios and function, our findings for the FDB may not represent the behaviour of other intrinsic (or extrinsic) foot muscles. In order to enable the collection of high-quality muscle fascicle images, the ultrasound transducer was fixed underneath the arch of the foot such that slipping of the transducer in any direction was largely eliminated. It is possible that pressure from the transducer against the sole of the foot may have influenced the natural motion of the foot and possibly the activation of the muscle. However, our measurements were within-subject comparisons, so any compensations made as a result of probe pressure would be present for all tasks measured. The shoe worn by all participants to protect the transducer and foot introduced additional compliance underneath the foot, as well as the possibility of altering external moment arms at the foot and ankle joints. While we chose a stiff foam to minimize any compliance and damping effects, the shoe probably dissipated some mechanical energy, which would be included in our UD foot power calculations.

Variations in human foot morphology and innervation are known to exist between males and females, which could result in differences in foot function during locomotion. Thus, it was intended to recruit equal numbers of female and male participants. However, because of recruitment restraints imposed by Covid-19, only a convenience sample was obtained and this sample did not have an equal balance of male and female participants. Of note, the similarities of human feet between sexes far outweigh their differences, particularly in regard to the anatomy of the foot's muscles and tendons. Therefore, we are confident in the results and interpretations of data in this work, albeit there may be some subtle differences as a result of sex, race or other attributes that we are not able to distinguish here because of the relatively small and homogeneous sample size.

In conclusion, we have demonstrated that the FDB utilizes its tendinous tissues to enhance power output during arch recoil when mechanical energy is produced at the foot, as well as to buffer power input into its muscle fascicles while the foot dissipates energy. This is the first *in vivo* evidence that the energy generation and dissipation capacities of the foot are modulated by the muscle and tendon dynamics of the intrinsic foot muscles and further highlights the importance of tendon compliance across human lower limb muscles for work generation and energy absorption.
